# Perceptual-cognitive skills in talent development environments: a survey of academy football coaches in the United Kingdom

**DOI:** 10.3389/fpsyg.2026.1751602

**Published:** 2026-03-10

**Authors:** Andrew O. Triggs, Joe Causer, Allistair P. McRobert, Matthew J. Reeves, Matthew Andrew

**Affiliations:** 1 UCFB Manchester Campus, Manchester, United Kingdom; 2 Research Institute for Sport and Exercise Sciences, Liverpool John Moores University, Liverpool, United Kingdom; 3 Department for Sport and Exercise Science, Manchester Metropolitan University, Manchester, United Kingdom; 4 Lancashire Research Centre for Applied Sport, Physical Activity and Performance, University of Lancashire, Preston, United Kingdom; 5 College of Sport Sciences and Physical Activity, Princess Nourah Bint Abdulrahman University, Riyadh, Saudi Arabia

**Keywords:** coaching, expert performance, game intelligence, scouting, soccer

## Abstract

**Introduction:**

Perceptual-cognitive skills (PCS) are a strong predictor of future expert performance in football. Theoretical and practical knowledge of PCS are important to inform (de)selection and development decisions. Despite their relevance to player development, limited research has explored how coaches working in talent development environments conceptualise and assess PCS in practice. This study aimed to critically examine perceptions of and engagement with PCS identification and assessment among UK academy coaches.

**Methods:**

An online survey collected data from 63 academy coaches regarding their understanding and identification of PCS within the age groups they coach.

**Results:**

Data indicated agreement between coaches PCS definitions and their importance in player development. However, variations in coaches’ familiarity, confidence, and frequency of PCS identification, alongside differing views on the importance of specific PCS point to potential knowledge and practice gaps.

**Discussion:**

Further research is needed to understand and develop interventions that support coaches in enhancing PCS identification and assessment within this overlooked area of player development.

## Introduction

The process of identifying talented youth football players, providing them with a bespoke environment and pathway that progresses them toward the first (i.e., senior) team and contribute toward their future success is at the forefront of many talent development systems (Relvas et al., 2010; Reeves et al., 2018b). To support the talent identification (TI) and development (TD) processes of professional football clubs, researchers have examined potential future predictors of adult expert performance such as physical (e.g., speed; Datson et al., 2020), skill (e.g., passing; Höner et al., 2019), sociological (e.g., developmental activities; Reeves et al., 2018a), psychological (e.g., grit, Larkin et al., 2023), and chance events (e.g., socio-economic status; Kelly et al., 2024).

An important predictor of future professional football performance are perceptual-cognitive skills (PCS; Jordet et al., 2020; Roca et al., 2013), which is a player’s ability to identify and acquire environmental information that is integrated with existing knowledge, in order to select and execute an appropriate goal-directed response (Marteniuk, 1976; Triggs et al., 2025a). PCS include a players ability to acknowledge familiar configurations (i.e., pattern recognition), predict what will happen next (i.e., anticipation) and select the most suitable action (i.e., decision-making), and are consistently shown to distinguish between future higher (e.g., professional) and lower levels of adult football performance (Scharfen and Memmert, 2019). PCS exhibited by higher skilled players are underpinned by more effective visual exploratory activities (VEA) characterized by frequent scanning (i.e., head turns directed away from the ball; Jordet, 2005) and efficient gaze strategies (i.e., the ability to focus visual attention to information-relevant areas during the game; Roca et al., 2011). For example, evidence from 11 vs. 11 match-play indicates that greater scanning frequency and exploration time prior to ball reception are associated with successful and progressive passing actions, adaptive body orientations, and faster responses under pressure, particularly among central midfielders and central defenders (Jordet et al., 2020; McGuckian et al., 2018, 2019; Pokolm et al., 2022). These behaviors vary as a function of task constraints such as opponent pressure, pitch location, and player role, reinforcing their relevance within competitive performance environments (Aksum et al., 2021; McGuckian et al., 2020). Furthermore, research utilizing representative task designs has highlighted higher skilled players often utilize more eye-movement fixations of shorter duration and toward a greater number of locations in the display (i.e., opponents, teammates, and areas of free space; Roca et al., 2011, 2013). PCS are therefore important for TI and TD in football (O’Connor et al., 2016; Ward and Williams, 2003; Williams, 2000) and, thus, coaches who support the systematic development of players must be equipped to identify, assess, and develop these skills (Larkin and Reeves, 2018).

Coaches are key decision-makers within academy football TD environments, routinely tasked with making (de) selection decisions and designing development programs based on their perceptions of potential (Unnithan et al., 2012). Therefore, it is important they possess an understanding of areas associated with TI and TD (e.g., technical/skill; tactical; psychological; sociological; physical) with the risk of overlooking key predictors of future performance or misjudging a player’s developmental trajectory if they do not (Williams et al., 2020). There is a plethora of evidence examining the mechanisms that support improved PCS performance in football from players’ perspective; however limited attention has been given to how coaches understand and engage with this component of TI and TD (Reeves et al., 2019; Roberts et al., 2019; Triggs et al., 2025a). Previous literature has highlighted that PCS, such as decision-making, anticipation, and awareness are highly rated attributes by practitioners (e.g., coaches, scouts) when making identification and selection decisions in Europe, Australia and the United States (e.g., Andrew et al., 2025a; Bergkamp et al., 2022; Christensen, 2009; Fuhre et al., 2022; Larkin and O’Connor, 2017) and across playing positions (Roberts et al., 2019). While the importance of PCS for future performance is widely endorsed by coaches, investigations reveal variability in how these skills are conceptualized, defined, prioritized and operationalized by coaches in applied settings (O’Connor et al., 2018; Pulling et al., 2018; Triggs et al., 2025b). Qualitative studies within academy environments has shown coaches advocate the importance of decision-making and VEAs for performance, yet they have uncertainties regarding how they should be explicitly developed, integrated, and evaluated in practice (Eldridge et al., 2023; Maskell et al., 2024; Triggs et al., 2025b). These findings suggest that although certain components of PCS expertise are highly valued in principle, coaches’ applied engagement with PCS differs and is shaped by experience, education, and contextual constraints (Pulling et al., 2018; Triggs et al., 2025b). Despite increased interest in PCS-related constructs, there remains limited larger-scale evidence examining how practitioners in TD environments (e.g., academy coaches and scouts) conceptualize PCS, and how this understanding informs their identification and assessment practices within structured TD environments (Triggs et al., 2025b).

Without a clear and shared understanding of PCS, coaches risk becoming inconsistent and/or ineffective within their practices (Larkin and Reeves, 2018; Miller et al., 2015). For example, previous studies using systematic observation have shown that professional youth academy coaches of male players employ a high proportion of activities within their practice that are not consistent with the scientific literature for optimally developing PCS (e.g., Andrew et al., 2021; Ford et al., 2010; Williams and Hodges, 2005). Moreover, qualitative interviews have revealed a lack of consistency and structured approaches regarding approaches to PCS identification and assessment in academy coaches in the United Kingdom (UK; Triggs et al., 2025b). These studies have typically been case study designs (i.e., single club) and/or have small sample sizes (Eldridge et al., 2023; Maskell et al., 2024; Triggs et al., 2025b), thus it currently remains unclear whether these findings and inconsistencies surrounding PCS perceptions and conceptualizations reflect broader trends across the academy system in the UK. Specifically, there is limited large-scale evidence capturing how academy coaches conceptualize PCS, the relative importance they attribute to different PCS, and their perceived approaches toward PCS identification and assessment. Addressing this gap is critical for establishing a shared understanding of PCS that may inform coach education, assessment practices, and future intervention design within academy football (Triggs et al., 2025b). A method previously utilized to gather system-wide perspectives from academy coaches operating across different clubs, development phases, and academy categories within the UK are online surveys (Alder et al., 2024; Kite et al., 2022; Roberts et al., 2019). This method enables access to a geographically dispersed and professionally embedded population, while facilitating standardized data collection across respondents in a time-efficient and scalable manner (Andrew et al., 2025a; Ford et al., 2023). Therefore, the aim of the present study was to examine how coaches working within professional youth football in the UK conceptualize PCS and their perceptions regarding the identification and assessment of these skills in their practice via an online survey.

## Methods

### Sampling

A purposive sampling approach was adopted due to its alignment with the specificity of the research question and the need for insights from a targeted population (Campbell et al., 2020). Participants could be full- or part-time age group football coaches, or phase lead coaches across the foundation- (FP; U9-U11), youth-development- (YDP; U12-U16), and professional-development-phase (PDP; U18-U23) within a category 1–3 (tiered system within Premier League framework) football academy in the UK. Individuals in closely connected roles (e.g., Head of Academy) were excluded as they were not directly involved in coaching delivery to specific groups on a regular basis. From 86 eligible clubs, a total of 150 stakeholders were contacted across category 1 (*n* = 66), 2 (*n* = 27), and 3 (*n* = 57) academies. Other staff members (e.g., Academy Manager; Head of Coaching) were requested to share the survey link to support participant recruitment, creating a snowball effect (Palinkas et al., 2015). Direct outreach was conducted via the research team’s industry network, using email and social media platforms (i.e., LinkedIn; X). To extend recruitment, a targeted recruitment infographic was created and shared with the aim of reaching additional eligible participants through snowballing impacts of re-posts, likes, and shares (McRobert et al., 2018). The survey opened on 7th August 2023, and closed on 30th May 2024, covering the entire 2023–24 UK football season.

### Materials and procedure

An online survey (JISC v2, Bristol, United Kingdom) was used to capture coaches’ conceptualizations (i.e., how coaches define and understand PCS as a construct) and perceptions (i.e., coaches’ subjective evaluations and judgments) of PCS and their engagement with the identification and assessment of these skills. Survey questions were deductively informed by the extant literature on PCS and TD designed to reflect key constructs commonly examined within research (e.g., understanding, importance of characteristics, and assessment practices; Patton, 2002). In addition, insights from recent qualitative work with academy coaches (Triggs et al., 2025b) were used to refine questions and ensure contextual relevance. Survey topics included: (1) coaching background (e.g., qualifications; experience); (2) understanding and familiarity with PCS; (3) perceived importance, frequency, and confidence identifying and assessing PCS; (4) importance rating of specific PCS (e.g., scanning, anticipation, decision-making); and (5) PCS specific education. For the importance ratings of individual PCS, definitions, descriptions, and looping video clips were provided to standardize terminology and ensure participants were reflecting from a common reference point (Table 1; Lewandowski, 2008).

**Table 1:** PCS definitions provided to participants.

Skill	Definition	Adapted from
Scanning	Active head movement where a player briefly redirects their vision away from the ball to gather information and assess the surrounding game environment. It helps players maintain awareness of the field and make informed decisions.	Jordet et al. (2020)
Gaze strategies	The way players use their visual attention and eye movements to gather information and make decisions on the field. It involves directing focus to specific areas and objects, as well as the timing and sequence of gaze shifts.	Button et al. (2011)
Anticipation	Ability to predict the actions and movements of teammates, opponents, and the ball. It involves reading the game and making proactive decisions based on expectations.	Williams and Jackson, 2019
Decision-making	Ability to analyze information, assess various options, and select the most appropriate action in a given situation.	Memmert and Roca (2019)
Pattern recall	Ability to recognize and remember recurring patterns or sequences of play on the field. It involves quickly recalling previously observed patterns and making informed decisions based on that knowledge.	van Maarseveen et al. (2015)
Creativity	Ability to generate novel and innovative solutions or actions in challenging and unpredictable situations. It encompasses thinking outside the box and finding unique ways to solve problems on the field.	Roca et al. (2021)

Prior to data collection, the survey was sense-checked across the research team before being pilot tested by an Academy Manager of a Category 3 football academy. They possesed a Union of European Football Associations (UEFA “A”) coaching license and over 15 years coaching experience. Following the pilot, an informal interview was conducted to refine the survey such as phrasing, duration, and media suitability (no data from the pilot was included in the final analysis). Survey completion time was prioritized given the time constraints associated with the profession of the target population (Buhrmester et al., 2011; Rice et al., 2017). Revisions were implemented, confirmed with the pilot participant for clarity before a final review by the research team, who have experience with survey-based research (e.g., Andrew et al., 2025a; Roberts et al., 2019). The final survey contained 19 questions (coaching background; *n* = 8, PCS specific; *n* = 1 open-ended; 5 multiple-choice; 6 ranking) across four sections. Likert scale responses were on a 5-point scale and ranking responses were out of 6 with points labeled with qualitative anchors (Wade, 2006). Upon accessing the survey, participants reviewed the participant information sheet; and selecting “continue” indicated informed voluntary consent. Anonymity was assured for all participants. Ethical approval was obtained from the lead institution (23/SPS/046).

### Analysis

Quantitative data were exported from the survey platform into a Microsoft Excel (Microsoft, Washington, United States) csv file for analysis. The study adopted an exploratory, descriptive approach aimed at capturing academy coaches’ perspectives on PCS. Accordingly, no *a priori* power calculations were conducted, as the analyses did not involve hypothesis testing or inferential comparisons. Within this context, the achieved sample size (*n* = 63) provides a considerable starting point for providing initial system-wide insight of academy coaches’ conceptualizations and perceptions of PCS within the UK. Although modest relative to the total number of coaches operating across the academy system, this sample reflects the practical realities of recruiting from a professionally embedded and time-constrained population (O’Gorman et al., 2021). Research involving academy coaches frequently reports smaller samples (e.g., Roca and Ford, 2020, *n* = 53; Nosek et al., 2021, *n* = 34; Page et al., 2025, *n* = 34; Pulling et al., 2018, *n* = 58) often due to competing demands, limited availability, and low engagement with research activities (O’Gorman et al., 2021). The sample for this study represents one of the largest datasets to have examined academy coaches’ perspectives on PCS; a topic that has received limited empirical attention at scale (Triggs et al., 2025a). Consequently, the findings should be interpreted as indicative of prevailing coach perspectives rather than statistically representative estimates of the wider population (De Vaus, 2013).

Coaches’ definitions of PCS from the open-ended question were thematically analyzed by generating codes to capture key segments within each definition. MindNode was used to visually map links among these codes and explore potential thematic structures (Braun and Clarke, 2006, 2019). The research team reviewed and refined codes and themes, enhancing criticality through iterative discussion to ensure themes were supported by sufficient data depth and breadth (Braun et al., 2017; Smith and McGannon, 2018). Through this iterative process, themes were named and finalized (Braun and Clarke, 2006). Frequency analysis was conducted for categorical and multiple-choice questions, with the percentage of respondents reported for each response. Likert scale responses were converted to integers and represented by the qualitative anchor associated with the mean response (Hopkins, 2010).

## Results and discussion

The present study utilized an online survey to examine how academy coaches in the UK conceptualize PCS, and their perceptions regarding the identification and assessment of these skills. In total, 63 coaches participated (male = 62, female = 1), and worked with players aged between 8 and 18 years covering all development phases (Table 2). Coaches demonstrated a shared definition of PCS, and acknowledged their importance for TD. However, variation was observed in coaches’ familiarity with PCS and in their prioritization of specific PCS. These are examined in relation to coaches’ reported frequency and confidence in identifying and assessing PCS, and the role of coach education and development activity in shaping coaches’ engagement with PCS. Findings are discussed with a focus on implications for TD processes and recommendations for future research.

**Table 2:** Participant characteristics.

Characteristic	Category	Mean (±)
Age (years)		35.5 ± 8.5
Coaching experience (years)		15.7 ± 7.1
Academy experience (years)		9.6 ± 6.6
		*n (%)*
Highest coaching qualification(s)^1^	UEFA Pro (Level 5) UEFA A (Level 4) FA ‘AYA’^2^ (Level 4) UEFA B (Level 3) UEFA C (Level 2)	2 (3) 29 (46) 13 (21) 23 (37) 2 (3)	Coaching qualification (in progress)	FA ‘AYA’^2^ (Level 4) UEFA B (Level 3)	16 (25) 2 (3)
Academy status	Category 1 Category 2 Category 3	32 (51) 7 (11) 24 (38)
Age group/s coached^3^	U9 U10 U11 U12 U13 U14 U15 U16 U18	20 (32) 18 (27) 18 (27) 18 (27) 15 (24) 19 (30) 12 (19) 16 (25) 11 (18)

^1^Some participants held both level 4 qualifications (i.e., UEFA A-License and Advanced Youth Award) and/or coached multiple age groups, meaning the total percentage exceeds 100%.^2^AYA refers to the Football Association’s Advanced Youth Award, launched in 2011.^3^In UK academies, U9-U12 consists of foundation phase (FP) players, U13-U16 consists of youth development phase (YDP) players, and U18 -U21 consists of professional development phase players.

### Defining PCS

Agreed definitions of key terms within TD are crucial to ensure consistent interpretations among coaches, supporting greater uniformity and efficacy in applied work (Baker et al., 2018, 2024; Johnston et al., 2023). Ambiguities around terms such as “talent,” “potential,” and “gifts” in TI and TD contexts can create doubt, leaving coaches unclear about what certain attributes look like, act like and develop into (Baker et al., 2024). For PCS, however, the thematic analysis highlighted coaches were largely consistent in their definitions, frequently emphasizing *processing information* from the environment and *making effective decisions*. The consistency of the definition of PCS by high-level football coaches differs from previous reports (O’Connor et al., 2018) and is line with original definitions (Marteniuk, 1976). This may be due to differences in the populations recruited, where the absolute playing level (i.e., highest levels of youth football in the United Kingdom) was unclear in previous studies (McAuley et al., 2022; O’Connor et al., 2018). While the phrasing of PCS definitions varied among participants, the underlying theme of what PCS are is encouraging from a TD perspective, as it suggests a shared understanding, and increases the likelihood of greater reliability in TD if stakeholder definitions have appropriate agreement (Baker et al., 2024).

### Importance of PCS

All coaches agreed on the importance of PCS within player development (see Table 3). This aligns with findings with TI and TD stakeholders in locations such as the Netherlands (scouts; Bergkamp et al., 2022), Australia (youth coaches; Larkin and O’Connor, 2017), Denmark (national team coaches; Christensen, 2009), the United States (college coaches; Andrew et al., 2025a), and builds on the insights within the UK, that has failed to provide coaches a voice (Reeves et al., 2019; Roberts et al., 2019). These perspectives align with the demands of football, where PCS play a positive role in English Premier League players’ performance, and are increasingly important as players progress through talent pathways (Aksum et al., 2021; Jordet et al., 2020). Laboratory-based research also underscores high skilled players’ decision-making, anticipation (Roca et al., 2011, 2013), pattern recognition (Williams et al., 2012), and VEA (Roca et al., 2018). Thus, an agreement between research and coaches in definitions and value for PCS should facilitate integration into applied practice. However, it is important to acknowledge the likely self-selection bias in the sample, as coaches who chose to complete the survey may already hold strong PCS interest. Thus, the high level of agreement observed may not reflect the full spectrum of views within the wider coaching population.

**Table 3:** Mean (±) responses to Likert-scale items (1–6).

Topic	Mean (±)
PCS familiarity	(2.94 ± 0.97) Moderately familiar
Importance of PCS	(4.62 ± 0.49) Very important
Observation and assessment of PCS	(3.73 ± 0.78) Frequently
Confidence observing and assessing PCS	(3.49 ± 0.76) Moderately confident
Prioritization of PCS in player development	
Scanning	2.27 ± 1.35
Gaze strategies	4.63 ± 1.37
Anticipation	3.49 ± 1.15
Decision-making	2.03 ± 1.09
Creativity	3.33 ± 1.42
Pattern recall	5.24 ± 1.07

Engaged in PCS training or education	% (No.)
Yes	36.5 (23)
No	63.5 (40)

For familiarity, importance, observation and assessment, and confidence, higher mean values indicate greater familiarity, importance, frequency, and confidence (1-5). For the prioritization of individual PCS, lower mean values indicate higher prioritization (1 = most important; 6 = least important). The table also reports the proportion of coaches engaged in PCS-specific training or education (%).

Views on PCS presented by coaches may represent a change in attitudes and approaches toward TD processes in the UK. Historically, physical and physiological metrics have been emphasized in academy football, with physical testing and profiling requirements within academy frameworks in the UK (Premier League, 2011). Indeed, recruitment of staff specializing in physical testing and development (i.e., strength and conditioning coaches, sport scientists) has made physical profiles at the forefront of many processes (Page et al., 2025). Yet, growing awareness of biases associated with physical advantages such as the Relative Age Effect (i.e., an overrepresentation of players born closer to a “cut-off” date compared to players born later in that same year) and maturity (i.e., progression toward the mature adult state) bias (Andrew et al., 2022, 2025b; Dugdale et al., 2021; Kelly et al., 2020) may shift coach focus. This evolving understanding could reflect a change in coaches’ attitudes toward PCS, highlighting the importance of perceptual and cognitive skills, even when resource allocation continues to prioritize physical development. Coaches’ views suggested that a broader, more holistic approach to TD may be emerging; one that can balance both physical and perceptual-cognitive attributes within player development (Williams et al., 2020).

### Familiarity

Coaches familiarity with PCS demonstrated a somewhat standard distribution from “not” to “very” familiar, where coach ratings contrasted with their perceived importance and definitions of what PCS are. One explanation for this could be that coaches have declarative knowledge (i.e., awareness of understanding; definitions and recognition of terms), but lack the procedural knowledge (i.e., practical application) of to how to incorporate these skills into their practice (Abraham and Collins, 1998, 2011). Only 3% of coaches stated they were “very” familiar with PCS. Coaches in the present study are working in high-level, professionalized football TD environments in the UK. Therefore, it could be expected that they would be “very” familiar with all components of player development (Côté and Gilbert, 2009; Partington et al., 2014). However, with the nature of academy football, some staff are part-time and so the varying qualifications and experiences could explain the range of answers regarding familiarity. Previous research has shown experience and qualifications supports engagement and attitudes toward VEA training (Pulling et al., 2018). However, coaching experience has been shown to have no significant association with the interpretation of technical and tactical football performance against objective data when observing small-sided games (O’Brien-Smith et al., 2024). As a result, it was unclear whether experience and qualifications impacted participants familiarity with the term PCS and what specifically they are less familiar with.

A further potential explanation for the varied self-reported familiarity scores was how terminology might have been interpreted by coaches. Within TD environments, terminology is often used inconsistently, and coaches may be more accustomed to alternative labels such as “game intelligence,” “game IQ,” “game understanding,” or “game sense” (Fortin-Guichard et al., 2025; Johnston et al., 2023). While these terms are frequently used interchangeably, their meaning may differ substantially between individuals and clubs. For instance, some coaches may interpret them as referring primarily to tactical skills and/or decision-making, whereas others may include perceptual elements such as visual search behaviors. These potential variations in interpretation have important practical implications, as conceptual ambiguity can hinder effective communication between coaches, reduce alignment across multidisciplinary teams, complicating the identification, assessment, and development of PCS. Moreover, when terminology is interpreted differently across contexts, coaches may believe they are targeting similar constructs while, in practice, focusing on different skills and behaviors. This may partially explain discrepancies in familiarity reported across respondents and highlights the importance of using language that is both accessible and precise when engaging coaches in research and applied practice (Triggs et al., 2025b). Establishing clearer, shared definitions, whether referring to PCS explicitly or to commonly used alternative terms may support more consistent understanding and improve the translation of research into practice within TD environments (Johnston et al., 2023).

PCS engagement within coaches’ day-to-day practices could have impacted their perceived familiarity with PCS. Previous research utilizing academy coaches highlighted the varying engagement and difficulty incorporating and developing VEA within training sessions (Eldridge et al., 2023). Football academies have their own unique philosophies and values that defines their TI and TD processes and approaches, that are not governed by the philosophy of national associations (Ford et al., 2023; Premier League, 2011). These mixed philosophies could result in varied exposure clubs provide to PCS (Triggs et al., 2025b). Key stakeholders such as Academy Managers are responsible for the creation, development, and implementation of the clubs TD program which may incorporate high quantities of technical, unopposed practice activities that attenuate coaches opportunities to identify, assess, and develop PCS (Andrew et al., 2021; Ford et al., 2010). Therefore, it is important to understand the context within individual clubs and the impact this might have on coaches engagement with this area of player development.

### Prioritization

While the importance of PCS was recognized by coaches, there were notable differences in their prioritization of specific PCS. This variability might have reflected the limited understanding of specific attributes compared with the more global term. Despite being provided with definitions, descriptions, and illustrative videos, some aspects of PCS may still be unfamiliar to coaches, indicated by their self-reported familiarity ratings. An example highlighting the potential gap in understanding was the contrasting prioritization of scanning versus gaze strategies, as 63% of coaches ranked scanning among their top two highest rated, whereas 63% placed gaze strategies as their lowest rated. The high prioritization of scanning aligns with existing literature, where coaches highlighted scanning as a key skill to develop from a young age (Eldridge et al., 2023; Pulling et al., 2018). However, scanning and gaze behaviors are closely connected, with scanning involving limited fixations (Aksum et al., 2021) and gaze strategies in dynamic environments involving shorter, more frequent fixations within a player’s field of vision among high level players (Roca et al., 2011). This disconnect regarding gaze strategies and scanning may be attributed to complexity of academic terms in PCS research, such as “fixations,” “quiet eye,” and “saccades,” which are less accessible to coaches (Piras et al., 2021; Vaeyens et al., 2007; Zhou, 2021). Moreover, gaze behaviors has been examined within laboratory-based environments, and very rarely *in situ*, thus lacking ecological validity (Dicks et al., 2010). As a result, coaches may not fully comprehend this terminology, or buy in to the transferability of research findings, especially given that typical coach education does not include this level of technical information (Dempsey et al., 2024). Developing accessible and transferable information on PCS in TD environments may be required to improve coaches’ knowledge of PCS.

### Identification and assessment

Coaches reported moderate to high confidence (92%) in their ability to identify and assess PCS, where 75% indicated they frequently or very frequently observe and assess these skills in their players. These data contrasted with only 3% of coaches who reported a high familiarity with PCS, raising how such a high level of confidence can coexist with limited familiarity. It may be that coaches have interpreted the survey questions based on the areas of PCS in which they felt most comfortable. As PCS encompass a broad range of perceptual and cognitive functions (Williams and Ericsson, 2005), it is plausible to suggest that coaches were more familiar with specific components, such as decision-making, which they might evaluate based on players’ alignment with their own interpretative frameworks (Barraclough et al., 2022; Lyons et al., 2011; McCormack et al., 2022). This narrower scope of understanding could contribute to the reported confidence levels, as coaches may feel assured within the limits of their existing knowledge (Stodter and Cushion, 2014).

The potential overconfidence phenomenon between coaches’ confidence and familiarity with PCS may also be understood through the lens of intuitive and tacit decision-making in coaching practice (Nash and Collins, 2006). Coaches frequently rely on rapid, experience-based judgments, often described as “gut feeling” when making evaluative decisions in complex and time-pressured environments like football (Lyle and Cushion, 2010). Within such contexts, confidence may emerge from repeated exposure to similar situations and the perceived effectiveness of intuitive judgments, rather than from explicit engagement with evidence-based approaches (Cushion et al., 2003). Coaching has also been described as an art form, with decisions driven by instinctive feel rather than articulated reasoning (Jones et al., 2004). While this experiential approach can be highly functional, it is often underpinned by tacit knowledge that is difficult to verbalize or formally justify (Nash and Collins, 2006; Sternberg and Horvath, 1999). As a result, coaches may struggle to explain why particular judgments are made or how specific performance attributes (such as PCS) are identified and assessed. Therefore, reliance on tacit and intuitive knowledge may lead to high confidence in evaluative ability while simultaneously obscuring gaps in explicit conceptual understanding. This may lead to assessments that are internally consistent within individuals’ coaching frameworks but inconsistently aligned with shared practice across coaching environments (Barraclough et al., 2024; Triggs et al., 2025b).

### Education

Only 37% of coaches reported having previously received any specific PCS education. Among the varied responses describing this education, the only recurring education stated was a Level 4, Football Association course that is exclusively available to those working within the academy system. This aligned with previous interview findings with academy coaches, highlighting the same course as a predominant source of information regarding PCS (Triggs et al., 2025b). The limited formal education in PCS among 63% of coaches may underpin discrepancies between their reported confidence and actual familiarity with PCS. Coaches may exhibit an inflated sense of confidence based on their existing but limited knowledge, often informed by traditional practices rather than evidence-based approaches (Andrew et al., 2021; Cushion et al., 2003; Williams and Hodges, 2005, 2023). While coaches express a desire to create decision-making environments, they often lack the expertise to effectively implement such practices (Partington and Cushion, 2013). This epistemological gap may also apply to PCS, where coaches recognized their importance but remained uncertain about their application in practice due to insufficient education (Light, 2008). Thus, minimal engagement with PCS-specific training, combined with the restricted availability of structured educational opportunities, likely contributed to the observed gaps in PCS familiarity, particularly among coaches without access to advanced courses.

### Limitations

There was an inability to differentiate between full- and part-time coaches in this study. However, while employment status may influence daily contact time and formal responsibilities, it does not necessarily reflect coaching experience, expertise, or qualification level. Within the competitive and resource-constrained academy environment, part-time coaches may possess comparable or greater formal qualifications and applied experience than full-time staff, reflecting the high workload demands and fluid employment structures characterizing youth football in the UK (Baker et al., 2025). Consequently, interpreting differences in PCS perceptions based on employment status may risk oversimplifying the relationship between role and applied knowledge. A further limitation of the study concerns the classification of coaches by age group or development phase. Although such categorization may appear conceptually straightforward, coaching roles within academy football are often fluid. In practice, coaches may operate across multiple age groups, transition between development phases, or follow player cohorts longitudinally over several seasons (Relvas et al., 2010). As a result, attempts to categorize coaches into discrete age- or phase-based groups may oversimplify their roles and obscure meaningful variation in how PCS are prioritized, identified, and assessed across developmental contexts.

### Practical implications and future directions

Understanding current applied practices, including football coaches’ knowledge of PCS, and their application of this knowledge (or lack thereof) offers academies valuable opportunities for targeted interventions, such as coach educational programs, upskilling staff, and fostering consensus on standardized approaches to PCS within TD. This study highlighted a broad definitional agreement regarding PCS but also revealed uncertainty surrounding their specific components. To address this, ensuring that coaching staff possess both declarative knowledge (understanding what PCS are) and procedural knowledge (understanding how to apply them in practice) is crucial to improve TI and TD processes (Abraham and Collins, 2011). For example, education could incorporate applied workshops that link research-informed information on PCS to video-based examples, observational frameworks, and guided discussion, enabling coaches to translate abstract concepts into observable behaviors. In addition, reflective learning approaches such as collaborative discussion, shared observation tasks, and alignment exercises across coaching staff may help surface tacit assumptions, promoting more consistent interpretations of PCS within academies (Triggs et al., 2025b).

To build on these findings, several future research directions are proposed. First, further examination of the specific processes and methods coaches and other stakeholders (e.g., scouts) use to identify and assess PCS are essential for contextualizing current practices. The use of *in-situ* measures may provide insights into how PCS are integrated into TD. Specifically, investigating how coaches currently identify and assess PCS in practice through measures such as eye tracking technology and verbal report protocols (e.g., Aksum et al., 2021; Roca et al., 2011) may provide deeper insights into these gaps and inform tailored training programs to bridge them. Moreover, exploring how factors such as coaches’ experience, qualifications, employment status, age and phase coached, and the format or depth of PCS-related education influence coaches’ engagement with PCS could inform the design of tailored educational strategies. Such insights may help ensure that all coaches, regardless of their background, have a robust understanding of PCS when working within TD environments. Finally developing practical tools and frameworks to enhance coaches’ and scouts’ ability to understand and assess PCS in applied settings represents a promising area for exploration. These tools may support stakeholders in recognizing a broader range of PCS and understanding how these skills manifest in different contexts, thereby improving both familiarity and application. By addressing these gaps, future efforts can contribute to a more consistent and evidence-informed approach to PCS in football.

## Conclusion

The present study highlighted that UK academy football coaches perceive PCS as important in player development, consistent with previous research (Christensen, 2009; Larkin and O’Connor, 2017). However, coaches reported varied familiarity with PCS, yet simultaneously expressed high confidence and frequency in identifying these skills within their practices. This discrepancy suggests potential issues that may compromise TI and TD processes over time if coaches lack sufficient knowledge, yet believe they can accurately assess and develop these skills. Further investigation into the education and applied practices when identifying and assessing PCS is essential to determine how additional support is needed to enhance these processes.

## Data Availability

The raw data supporting the conclusions of this article will be made available by the authors, without undue reservation.
